# Improving the Process of Shared Decision-Making by Integrating Online Structured Information and Self-Assessment Tools

**DOI:** 10.3390/jpm12020256

**Published:** 2022-02-10

**Authors:** Pei-Jung Hsu, Chia-Ying Wu, Lu-Cheng Kuo, Ming-Yuan Chen, Yu-Ling Chen, Szu-Fen Huang, Pao-Yu Chuang, Jih-Shuin Jerng, Shey-Ying Chen

**Affiliations:** 1 Center for Quality Management, National Taiwan University Hospital, Taipei 10002, Taiwan; pjhsu@ntuh.gov.tw (P.-J.H.); 120605@ntuh.gov.tw (C.-Y.W.); 119537@ntuh.gov.tw (Y.-L.C.); chuang@ntuh.gov.tw (P.-Y.C.); erdrcsy@ntu.edu.tw (S.-Y.C.); 2 Department of Internal Medicine, National Taiwan University Hospital, Taipei 10002, Taiwan; kuolc@ntu.edu.tw; 3 Office of Information Technology, National Taiwan University Hospital, Taipei 10002, Taiwan; mychen@ntuh.gov.tw; 4 Department of Nursing, National Taiwan University Hospital, Taipei 10002, Taiwan; sfhuang0406@ntuh.gov.tw; 5 Department of Emergency Medicine, National Taiwan University Hospital, Taipei 10002, Taiwan

**Keywords:** shared decision-making, patient decision aids, digital patient–provider communication tool

## Abstract

The integration of face-to-face communication and online processes to provide access to information and self-assessment tools may improve shared decision-making (SDM) processes. We aimed to assess the effectiveness of implementing an online SDM process with topics and content developed through a participatory design approach. We analyzed the triggered and completed SDM cases with responses from participants at a medical center in Taiwan. Data were retrieved from the Research Electronic Data Capture (REDCap) database of the hospital for analysis. Each team developed web-based patient decision aids (PDA) with empirical evidence in a multi-digitized manner, allowing patients to scan QR codes on a leaflet using their mobile phones and then read the PDA content online. From July 2019 to December 2020, 48 web-based SDM topics were implemented in the 24 clinical departments of this hospital. The results showed that using the REDCap system improved SDM efficiency and quality. Implementing an online SDM process integrated with face-to-face communication enhanced the practice and effectiveness of SDM, possibly through the flexibility of accessing information, self-assessment, and feedback evaluation.

## 1. Introduction

Shared decision-making (SDM) has become a central element of patient-centered care. Research, policies, and clinical guidelines have strongly advocated the implementation of SDM. The SDM model is a clinical decision-making model that ensures that healthcare professionals do not make decisions solely based on knowledge, experience, and the latest scientific evidence, but also by allowing patients to participate in all essential aspects of the medical decision. Patients need and have the right to understand available treatment options and participate in decision-making regarding their health.

The objective of the SDM process is to find the best treatment for a specific patient by encouraging the patient to play a more active role in the process of medical consultation [1]. Thus, eliciting patient preferences is a vital component of SDM [2]. However, recent findings show that patient preferences cannot be efficiently or accurately judged based on communicative exchanges during routine office visits, even for patients seeking to expand their decision-making role [3]. Patient decision aids (PDA) are tools used to inform patients who want to actively participate in health decision-making and help them make clear choices. These tools are most effective when used together with counseling from a healthcare provider. They provide information about a health condition using the latest quality-rated scientific evidence and options and outcomes regarding the diagnosis and treatment of the disease. In addition, they help clarify patients’ values and understanding of the relative importance of the benefits and risks of these options.

In the digital era, many of the processes regarding patient-informed health decision-making may benefit from being available online, such as understanding the disease; knowledge of related healthcare alternatives, benefits, risks, and uncertainties; personal preferences and values; and participation according to the role. When applied to patient education and decision aids, “digital” usually refers to software and platforms for teaching and learning that can be used with video or audio players, computers, or mobile devices. An increasing number of digital consumer and patient health tools are being developed for use on electronic devices such as computers and smartphones as standalone software or websites. Online resources to facilitate SDM have been advocated [4]; however, concerns have been raised over the effectiveness of electronic assessment tools at improving the shared decision-making process [5]. Various techniques have been designed to help patients obtain the information they need to enable them to play an active role in their care under a more equitable partnership. Although SDM has been increasingly popular in healthcare practice in Asia [6] and Taiwan [7], barriers may still be encountered [8], and the integration of digital and online processes on a hospital-wide implementation scale has scarcely been reported. Leaders in healthcare organizations are keen to run their service more efficiently and respond to patients’ needs.

This study aimed to assess the effectiveness of integrating an online SDM process into existing practice with a participating design approach in a university-affiliated medical center.

## 2. Materials and Methods

### 2.1. Design and Setting

This was a retrospective analysis of our institutional electronic SDM process. The university-affiliated National Taiwan University Hospital (NTUH), part of the NTUH healthcare system, located in northern Taiwan, is a 2600-bed medical center with about 8000 employees, including 1400 physicians, that serves 9000 outpatients, 290 inpatient admissions, and 300 emergency patient visits daily. The Research Ethics Committee of the NTUH approved this study (RIN2021) and waived the need for informed consent from the participants.

### 2.2. Participatory Design Approach

**Figure 1:**
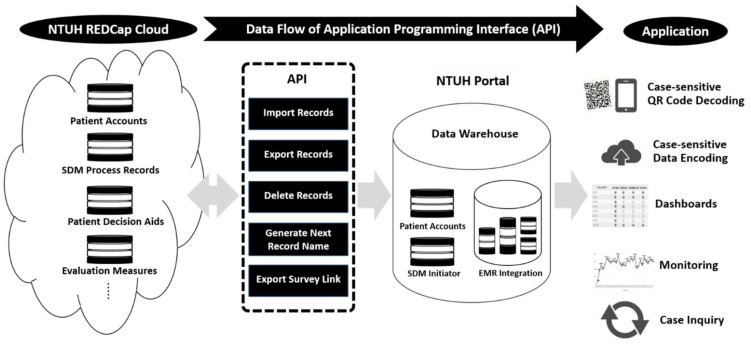
Schematic process of Research Electronic Data Capturing (REDCap) application in shared decision-making (SDM) management.

The current study is a qualitative study with a participatory design approach in which patients who need SDM, researchers, and system developers collaborated closely. Participatory research is the co-construction of research through partnerships between researchers and people affected by and responsible for action on issues of interest [9]. We used Research Electronic Data Capture (REDCap) as the primary strategy to form the infrastructure of the electronic SDM process, which was developed by the patients and teams from 24 clinical departments, the Information Technology Office, and the Center for Quality Management of NTUH. REDCap is a novel online methodology and solution developed by Vanderbilt University Medical Center to create and deploy electronic data capture tools to support clinical and translational research. REDCap is a secure, web-based software platform designed to support data capture for research studies, providing (1) an intuitive interface for validated data capture; (2) audit trails for tracking data manipulation and export procedures; (3) automated export procedures for seamless data downloads to common statistical packages; and (4) procedures for data integration and interoperability with external sources [10]. Data collection informed the development process and was guided by existing research on SDM. The final tool was developed based on REDCap and involved integrating an existing patient portal. See Figure 1 for an overview of the REDCap application in the SDM process.

### 2.3. Participants, Data Source, and Measurements

From July 2019 to December 2020, responses to the electronic version of SDM triggered by the clinicians were screened for their eligibility for the study.

We included response records of the online initiation of SDM processes with completed records of the first two SDM talks, i.e., choice and option talks, during the study period. Pertinent data were retrieved from the REDCap database of the Healthcare Information System (HIS) of NTUH. The following data were collected: topic, date, time, location, and department where the SDM process was triggered; participants in the face-to-face SDM discussion; age, gender, relationship to the patient of the respondent; concern assessments; tentative choice; and opinion about the PDA. As this study focused on the trend of SDM practices, we included all eligible responses, even if there were missing data regarding the characteristics or variables described above. We did not collect the number of potential SDM cases. We compared the numbers of cases and relevant information between the three 6-month periods involved in the study period. As the feedback evaluation tools differed across SDM topics preferred by the clinical teams participating in the design of SDM contents, including SDM-Q-9 [11], Preparation for Decision Making Scale (PrepDM) [12], SURE [13], and the customized questionnaires containing several items of question statements, the scores were standardized based on the ratio of the received total score to the allowed full score on each questionnaire type. The standardized evaluation score may allow for the intuitive interpretation of the data.

### 2.4. Statistical Analysis

We performed descriptive analyses of the characteristics of the SDM cases and respondents, the process data, and the trends of the cumulative number of electronic SDM cases from July 2019 to December 2020. Categorical variables were expressed with number and percentage; continuous variables with mean and SD. Comparisons between groups were performed using the chi-square test. We then performed multivariate linear regression analysis for the responding intervals and multivariate logistic regression analysis for the factors associated with decision preparedness. The analyses were performed using Microsoft Excel 2016 (Microsoft, Redmond, WA, USA) and STATA 15.0 (StataCorp LLC, College Station, TX, USA).

## 3. Results

### 3.1. Establishment of the Online SDM Structure and Process

The participatory approach in this program formed a developmental mechanism for the online SDM structure and process, providing a template for designing and establishing topics from the departments. The features of this online SDM are depicted in Figure S1 of Supplementary Materials, and include the following (see Figure S1 of Supplementary Materials):

- Displaying all established institutional SDM topics in the HIS to initiate and record the SDM process (Figure S1a).
- Documenting the SDM process in a structured format. Healthcare workers are guided to enter the necessary information, which is automatically transformed into records in the EMR (electronic medical records) (Figure S1b).
- Providing online information for the participants of SDM. A topic-specific, case-sensitive, QR code-containing print-out sheet or email is provided to every participant, who can access the information at any time after the face-to-face discussion (Figure S1c).
- Independently accessing online self-assessment tools through the QR code with a structured self-assessment tool to understand the patient’s clinical situation, options, matters, values, preferences, preparedness, and certainty for further talks with regard to decision-making (Figure S1d).
- Transferring feedback evaluation regarding the SDM process from the participants through the structured questionnaire based on the methods proposed in the literature [11,12,13,14] (Figure S1e).

### 3.2. Establishment of the Electronic SDM Topics

Table 1 summarizes the topics of SDM developed during the study period. The web-based PDA developed by each team with empirical evidence in a multi-digitized manner allowed the patients to scan QR codes on a leaflet using their mobile phones and then read the PDA content online. The patients could also share this information with other family members to make decisions together, and finally provide feedback on their preferences online. Each SDM manager/coach can also dynamically track the preferences of each case in REDCap and also obtain the feedback evaluation from the patients and surrogates online, saving time and improving management efficiency. From July 2019 to December 2020, 48 web-based SDM topics were implemented in the hospital. The system used for the SDM process consists of a smartphone QR code (for the patient) and a web portal (for the healthcare provider). Information entered into the web-based PDA by the patient or a family member is automatically transferred to the web portal, which can then be accessed by the coach and the healthcare team members.

### 3.3. SDM Processes and Completed Self-Assessment Responses

**Figure 2:**
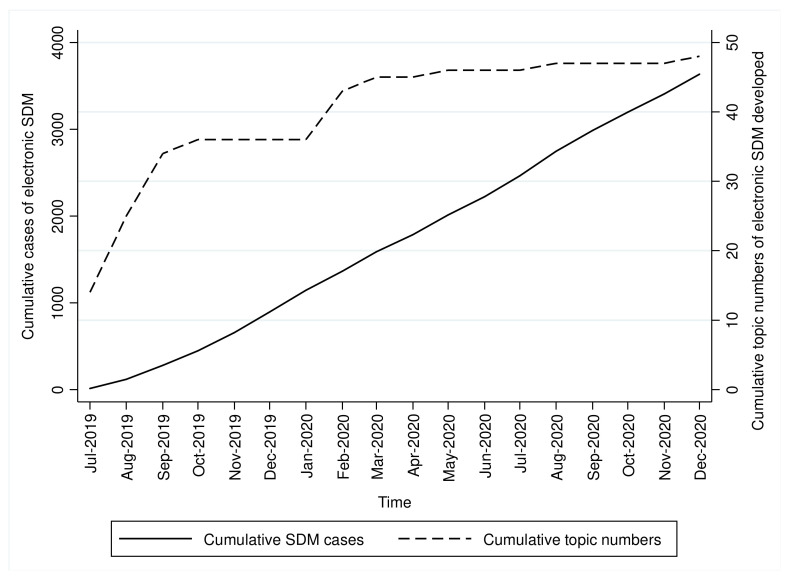
Cumulative numbers of implemented online SDM topics and cumulative SDM cases during the study period.

From the HIS, we identified 4145 cases with records of the online initiation of SDM processes with completed records of the first two SDM talks, i.e., choice and option talks, during the study period. Figure 2 shows the cumulative numbers of implemented online SDM topics and cumulative SDM cases during the study period. There was a progressive hospital-wide increase in the cumulative number of SDM cases. The cumulative number of SDM topics showed an approximate two-step increase. This was mainly due to the need for IT engineers to establish and test the online contents after a training course and workshop were provided to the SDM practitioners, and a consensus was reached after discussion toward the end of 2019.

Of the 4145 cases, 3756 (90.6%) had also completed PDA-assisted online assessments, with 3633 having an interval of no longer than 90 days between SDM initiation and response completion. We decided, therefore, to base the analysis on the data from these 3633 cases. Table 2 summarizes the information on the initiation of the SDM process. Patients from the departments of internal medicine (39.8%), family medicine (13.8%), and surgery (7.8%) accounted for most cases, and the outpatient setting was the most common (40.6%) location for the initiation of the SDM process. After the choice and option talks, the SDM teams assessed the patients or surrogates and found that 74% were highly likely to be ready for the decision talk (Table 2).

**Figure 3:**
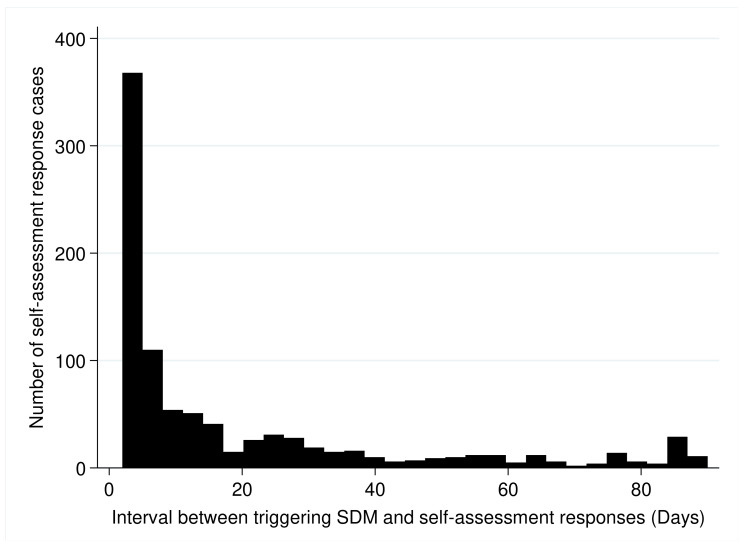
Distribution of intervals between triggering SDM and self-assessment responses in 933 cases of out-of-hospital self-assessments.

Comparisons among the three periods of six months showed a significant increase in mean monthly case numbers between each with documented completion of the SDM process and PDA assessment (149, 222, and 235 cases per month, ANOVA, *p* = 0.0203). The mean monthly completed SDM cases per topic were similar between the three periods (4.5, 5.2, and 5.0 cases, ANOVA, *p* = 0.6491). Table 3 summarizes the demographic features of the participants who provided online responses. Generally, they were middle-aged, and the number of male and female respondents was approximately equal. Nearly two-thirds of the respondents were the patients themselves, and the most common non-patient respondents were their children (Table 3). The respondents showed a rapid response time with an interval of 5.1 ± 0.2 days, even though a significant proportion of the responses were provided off-site. Of the 3633 cases, the timing of triggering SDM and completing self-assessment responses was on the same day in 2700 (74.3%) cases, suggesting that the self-assessments and responses of these cases were completed on-site in the hospital. For the 933 (25.7%) off-site cases, the response intervals are shown in Figure 3, which shows that the majority responded within three weeks after the initiation of the SDM process. Cases of surgical encounters were more likely (376 of 1031, 36.5%) to choose off-site responses than those of non-surgical encounter (557 of 2062, 21.4%) (*p* < 0.001). The percentages of cases choosing off-site responses were also different between outpatient (381 of 1048, 27.1%), inpatient (462 of 1353, 34.1%), and emergency department (27 of 747, 3.6%) care settings (*p* < 0.001). Comparisons among the three implementation periods showed that the trigger–response intervals were similar among the three periods (4.8 ± 0.5, 4.6 ± 0.4, and 5.8 ± 0.4 days, ANOVA *p* = 0.0843). In addition, 18 (0.5%) cases had more than one response; they were provided from different participants, including the patient and the surrogates.

The multivariate linear regression analysis, as summarized in Table 4, showed that several characteristics were associated with the response interval, with the patient as the respondent (coefficient = 2.168; *p* < 0.001) and surgical (vs. non-surgical) encounter (coefficient = 4.752; *p* < 0.001) significantly prolonging the interval, and emergency department encounter (coefficient = −4.754; *p* < 0.001) significantly shortening the interval. Gender and inpatient encounter (vs. outpatient) did not significantly affect the interval of online responses (Table 4).

Table 5 summarizes the multivariate logistic regression analysis results for the factors associated with the preparedness for decision-making. Factors including the patient as the respondent (OR = 3.480, *p* < 0.001), emergency department encounter (vs. outpatient) (OR = 24.963, *p* < 0.001), and inpatient encounter (vs. outpatient) (OR = 1.753, *p* < 0.001) were associated with reported preparedness for decision-making from the respondents, whereas gender, age, or surgical encounter (vs. non-surgical) were not associated with decision preparedness. Emergency department encounter was the most significant factor associated with reported preparedness for decision-making (OR = 24.963; CI = 12.122–51.407; *p* < 0.001) (Table 5).

Of the responses, 3169 provided feedback evaluation scores for the SDM process according to the experience of the participants. Of the designs of 48 topics, 23 had a Pre-DM questionnaire, 17 had SURE, and 12 had customized questionnaires established in the SDM process for the patient and surrogates to provide their feedback. The standardized score progressively increased throughout the three implementation phases (0.82 ± 0.01, 0.89 ± 0.01, and 0.89 ± 0.004, ANOVA *p* < 0.001). Table 6 summarizes the results of the multivariate regression analysis, which showed that multiple factors were associated with the scores, with patient-responder, age, surgical encounter, and emergency encounter being positively associated with the score, and gender and inpatient encounter being negatively associated with the score. Emergency department encounter was the most significant factor affecting the evaluation score (coefficient = 0.138; *p* < 0.001) (Table 6).

The results of clinicians’ feedback evaluations and responses are summarized in Tables S1 and S2 of the Supplementary Files. The responses suggested that this online integration of the SDM process saved time, especially for the SDM process. On the other hand, they showed the lowest score regarding the effectiveness of online integration in improving patient care (Table S1 of Supplementary File). Additionally, the clinicians placed high scores on the nine questions regarding their performance during the SDM process, with the lowest average scores being for “selecting option together” (5.0 ± 1.1) and “agree on how to proceed” (5.1 ± 1.0) (Table S2 of Supplementary File). Examples of interview responses are also summarized in the Supplementary File, with opinions generally positively supporting the integration of the online SDM process into the face-to-face process (see Supplementary File).

## 4. Discussion

In this study, we found that a significant proportion (about one-fourth) of the participants of SDM chose additional off-site assessments of the options and their preferences before expressing that they were prepared to make a decision rather than proceeding directly to on-site decision talks right after the option and decision talks. The provision of off-site case-specific online access to the SDM and PDA contents achieved a high response rate regarding the participants’ preparedness for decision-making. In addition, the output of the electronic SDM topics allowed for more than one response from the participants and their family to be captured. Therefore, the online self-assessment SDM process integrated with face-to-face communication enhanced the practice and effectiveness of the institutional SDM processes while preserving high response rates and achieving acceptable confidence when making decisions.

SDM processes involve face-to-face communication and discussions of the options for medical interventions or treatments throughout the three stages of the encounter, including the choice talk, options talk, and decision talk [15]. While this scenario provides direct explanation and feedback, the patients or their surrogates may face pressure to respond quickly before leaving the consultation. In addition, repeatedly introducing and explaining the decision needs and options may impose a burden on the SDM team. The decision quality may also be hindered by a suboptimal understanding of the options, the benefits and risks of the interventions, and the expression of personal matters, values, and preferences on such a fraught occasion. Therefore, our institutional approach of implementing online access for the SDM and PDA contents and assessment processes may provide a complementary mechanism for the patients and surrogates to more confidently prepare to make a decision. Furthermore, the patients, members of their family, and surrogates could also access the SDM and PDA contents at any time based on their needs for optimal understanding in order that they felt well-prepared to provide a response. We analyzed all the electronic SDM cases performed during the study period, and therefore we were able to describe the whole picture of the institutional implementation of the electronic SDM process and the trends of particular SDM topics.

Reports in the literature have shown the importance and benefits of online SDM tools, such as lung cancer screening [16], neurosurgical procedures [17], orthopedic interventions [18], and especially as a valuable adjunct to clinical discussions [19]. Online SDM tools have been reported to be cost-effective for patients with inguinal hernia, gallstones, and knee or hip osteoarthritis [20]. The online SDM and PDA services in our institution provide a full range of information and assessment tools coupled with real-time capturing of their responses, and this may allow clinicians of the SDM team to proceed with arranging the decision talk in a timely manner. The electronic process integrated with the usual face-to-face process provided a structured, guided approach for SDM. As clinicians might not be familiar with the concept and goal of SDM, they might need these structured processes and feedback from the patients, surrogates, and family members through the assessment forms. The real-time nature of the capture of online responses by the REDCap system allows SDM teams and clinicians caring for patients to receive real-time feedback and proceed in timely decision talks.

Although it is important to assess the effectiveness of the systematic implementation of this online process, in this hospital the development of online SDM and PDA contents and the integration of these contents into the REDCap platform required the active participation of the clinical department and healthcare workers in our hospital. Therefore, we considered that—since the online system has been optimized based on the needs of the clinicians—a before–after assessment by the participating clinicians might not be required. Nevertheless, our interviews with the clinicians provide their comments after the implementation of the online mechanism.

This report focused on the hospital-wide implementation of the online process by integrating it into the existing face-to-face process and thus a variety of SDM topics and practices from the departments of this hospital were involved. Therefore, the assessment of patient outcomes might be difficult as a result of a significantly mixed patient population and different clinician practices. Furthermore, the impact of SDM on clinical outcomes remained undetermined, whereas researchers also emphasized the measurement of patient-centered outcomes. 

In this study, we found a preference for off-site access to SDM and PDA compared with face-to-face practice among different patient and surrogate populations and care scenarios. Explanations include the severity of illness and urgency requiring timely decision making, the health literacy that might determine the speed and degree of understanding the options and preferences, the familiarity of digital and online platforms, and the number of participating persons for the decision in addition to the patient. We also found that age and gender might play significant roles in the online responses. Female responders tended to provide a lower score for the feedback evaluation of the SDM process, whereas older responders tended to require a longer interval to finish their online reviews and responses. These findings might reflect the cultural characteristics of Taiwan in terms of making decisions. These speculations require support from further research.

The implementation of the online SDM process in our institution highlighted that more time was needed to achieve preparedness for the final decision-making, suggesting the time-consuming nature of SDM. This is in line with other studies that have shown that SDM is more time-consuming but that patients may make better decisions [21]. In addition, providing sufficient time to re-assess options and preferences suggests that SDM is a more patient-centered approach [22]. Nevertheless, the implementation of SDM still faces multiple barriers [8]. Further integration of online content may be promising, and standardized videos could be considered [23]. Furthermore, scientifically analyzing the decision-making process may also be required in the future [24]. Healthcare professionals may also require better understanding and training on the SDM process and practice. Establishing an online SDM process may improve the completeness and correctness of practicing SDM in the real world [25]. The integration of artificial intelligence into PDA may also be a promising mechanism to enhance the effectiveness of SDM [26,27]. Nevertheless, challenges of SDM might exist, such as patients with multiple disease conditions and limited health literacy and older patients who require specially designed SDM tools [28], those lacking socioeconomic resources and family support, and the rapidly emerging management options for specific medical problems that demand timely updates for SDM, as well as healthcare settings facing the COVID pandemic [29]. Indeed, a versatile and convenient platform both for the providers and patients might be necessary for the era of digital transformation in the healthcare practice.

SDM has been an emerging and increasingly popular clinical practice in Taiwan, and several healthcare organizations have deployed online processes to help enhance the practice and outcomes. Nevertheless, since the National Taiwan University Hospital contributes to the REDCap community by translating Traditional Chinese language for REDCap, to our knowledge there was a lack of reports on the implementation of REDCap-based online SDM process that integrated into the face-to-face process for healthcare scenarios in Taiwan. The REDCap system applied in our healthcare system is a standardized server software that allows non-profit organizations to join the global REDCap consortium and to install and administer REDCap on their local servers in order to use for work at the organization. Therefore, the generalization of our model can be modified as a broader design approach with participants from different hospitals in Taiwan, involving the stakeholders and users from clinical departments, quality management, and IT engineers in the design process to co-design the online SDM integration and make sure the technical structure meets their needs and is applicable in their healthcare organizations. 

There are several limitations to this study. First, this study was based on analysis of records related to the electronic SDM process, while data related to the patients, such as diagnosis, comorbidities, interventions, and socioeconomic status, were lacking. Therefore, we did not know whether the patients received any of the interventions listed in the PDA contents to facilitate the SDM process. In addition, we did not know the exact time when the formal decisions were made, such as signing informed consent for interventions. Second, after completing the choice and option talks, we did not track whether there was additional contact between the SDM team and patients or surrogates. Therefore, whether the high off-site response rate to the online assessment process was due purely to the online process remains to be elucidated. Third, although the hospital-wide SDM processes were designed to be structural, we do not know how completely and correctly the SDM teams performed the choice and option talks for the patients and surrogates. Fourth, we do not have data on how often the patients and their surrogates accessed the online materials, nor did we know whether the responses were from single respondents or a collective opinion after a group discussion between family members and surrogates. Fifth, we did not access the clinical data to assess possible outcome changes brought by the implementation of the online process. This report focused on the hospital-wide implementation of the online process by integrating it into the existing face-to-face process, and thus a variety of SDM topics and practices from the departments of this hospital were involved. Therefore, the assessment of patient outcomes might be difficult as a result of a significantly mixed patient population and different clinician practices. Furthermore, the impact of SDM on clinical outcomes remains undetermined, whereas researchers have also emphasized the measurement of patient-centered outcomes. A variety of outcome assessments have been proposed for the evaluation of SDM processes [30,31,32,33]; therefore, the evaluation of this system can be further improved by establishing the measurements proposed by the experts and researchers. Last, this was a single center study. The generalization of our model and findings might need more robust designs, such as multicenter, randomized controlled trials [34], cluster randomized controlled trials [35], and multicentered, stepped wedge trials [20,36].

## 5. Conclusions

In conclusion, this study shows that a significant proportion of patients and their surrogates may benefit from off-site access to SDM and PDA information to allow them sufficient time to prepare for the final decision talk of the SDM process. Therefore, an online SDM process integrated with face-to-face communication may enhance the practice and effectiveness of SDM, while preserving a high response rate and acceptable confidence for making a decision. The flexibility of accessing information and self-assessment tools may provide additional benefits to enhance the value of personalized medicine.

## Figures and Tables

**Table 1:** Topics of SDM developed during the study period.

Topic	Topic
Long-term care settings for ventilator dependence	Options for smoking cessation
Tracheostomy for prolonged mechanical ventilation	The choice of hospice location
Choice of dialysis treatment	Treatment for traumatic rib fractures
Long-term nasogastric tube or gastric tube	Indwelling catheter for neurogenic bladder
Medications for poor oral hypoglycemic drug responders	Discharge preparation from rehabilitation ward
Choice of heart valves	Post-discharge care for elderly with reduced function
Treatment for severe brain damage	Post-stroke rehabilitation treatment
Intervention for heart failure with renal insufficiency	Rehabilitation after hip or knee fracture surgery
Reconstructing missing teeth	Treatment for poorly controlled atopic dermatitis
Choice of orthodontic device	Treating tuberous sclerosis & cutaneous angiofibroma
Treatment to assist upper jaw teeth pullback	Re-allocation after an occupational disaster
Method of obtaining head and neck tumor tissues	Integrated rehabilitative care for cancer patients
Hypothermia treatment after resuscitation	Treatment of children with urinary tract reflux
Follow-up medical care after first aid	Location of children’s end-of-life hospice
Wait in the emergency room or transfer	Management of teeth growth problem
Artificial joint replacement surgery	Nutrition for severe trauma/critically ill patients
Treatment for osteoporosis	Care for ventilator-dependent severe stroke
Integrated palliative care options for cancer patients	Radiation therapy for head and neck cancer
Use of unconventional sleeping drugs	Interventions for benign prostatic hyperplasia
Patient-controlled pain relief	Integrated psychological care for cancer patients
Treatment for Guillain-Barre syndrome	Integrated nutritional care for cancer patients
Treatment for high-risk metastatic prostate cancer	Integrated social worker management for cancer patients
Reproduction method for those at high risk	Integrated pain management for cancer patients
Treatment for advanced ovarian cancer	Multiple integrated care for cancer patients

**Table 2:** SDM cases for the analysis (*n* = 3633).

Characteristic	Data
Department	
Internal Medicine	1447 (39.8%)
Family Medicine	500 (13.8%)
Surgery	284 (7.8%)
Otolaryngology	281 (7.7%)
Psychiatry	268 (7.4%)
Dentistry	218 (6.0%)
Orthopedic	124 (3.4%)
Physical medicine and rehabilitation	87 (2.4%)
Medical Genetics	81 (2.2%)
Others	322 (8.9%)
Setting	
Outpatient	1476 (40.6%)
Inpatient	1404 (38.7%)
Emergency service	753 (20.7%)
Preparedness, evaluated by the SDM team	
Ready for decision-making	2015 (74.0%)
Not ready for decision-making	708 (26.0%)

**Table 3:** Characteristics of the respondents and SDM team (*n* = 3633).

Characteristic	Data
Respondents (*n* = 3633)	
Age, years	56.5 ± 0.3
Gender, male (%)	1883 (51.8%)
Relationship with the patient (*n* = 3606)	
The patient	2298 (63.7%)
Spouse	229 (6.4%)
Parent	240 (6.7%)
Child	742 (20.6%)
Sibling	94 (2.6%)
Other	3 (0.1%)
Interval between triggering SDM and response, days	5.1 ± 0.2
Online preliminary response for the choice	
Ready for decision-making	3208 (88.3%)
Not yet ready for decision-making	424 (11.7%)

**Table 4:** Multivariate linear regression analysis of the interval between triggering SDM and response (days) (*n* = 3482).

Variable	Coefficient	95% Confidence Interval	*p*-Value
The patient as the respondent	2.168	1.080–3.257	<0.001
Female	−0.267	−1.229–0696	0.59
Age	0.092	0.067–0.117	<0.001
Surgical encounter for SDM	4.752	3.587–5.918	<0.001
Emergency department encounter	−4.754	−6.086–−3.422	<0.001
Inpatient encounter	−0.349	−1.506–0.808	0.56
Constant	−1.740	−3.760–0.280	0.09

**Table 5:** Multivariate logistic regression analysis for the factors associated with preparedness for final decision-making (*n* = 3481).

Variable	Odds Ratio	95% Confidence Interval	*p*-Value
The patient as the respondent	3.480	2.715–4.459	<0.001
Female	0.825	0.665–1.025	0.08
Age	0.997	0.992–1.003	0.37
Surgical encounter for SDM	1.056	0.828–1.346	0.66
Emergency department encounter	24.963	12.122–51.407	<0.001
Inpatient encounter	1.753	1.372–2.240	<0.001

**Table 6:** Multivariate linear regression analysis for the feedback evaluation standardized score for the SDM process (*n* = 3049).

Variable	Coefficient	95% Confidence Interval	*p*-Value
The patient as the respondent	0.021	0.010–0.033	<0.001
Female	−0.038	−0.049–−0.028	<0.001
Age	0.001	<0.001–0.001	<0.001
Surgical encounter for SDM	0.025	0.012–0.037	<0.001
Emergency department encounter	0.138	0.123–0.153	<0.001
Inpatient encounter	−0.013	−0.026–<−0.001	0.04
Constant	0.821	0.799–0.843	<0.001

## Data Availability

The data supporting the reported results can be found at https://doi.org/10.5281/zenodo.5830050 (accessed on 19 January 2022).

## Supplementary Materials

The following supporting information can be downloaded at: https://www.mdpi.com/article/10.3390/jpm12020256/s1. Supplementary File (PDF), containing Table S1: Physicians’ response to online integration of the SDM process, Table S2: Clinicians’ evaluation of the whole SDM process, and Figure S1: Features of the online SDM process. (a) Display of all topics in the HIS for triggering SDM. (b) Structured records of the SDM process. (c) Online information for the participants of SDM. (d) Self-assessment of the patient/surrogates. (e) Feedback evaluation for the SDM process.
