# Supplementary material for: Improving the Process of Shared Decision-Making by Integrating Online Structured Information and Self-Assessment Tools
Source: J Pers Med. 2022 Feb 10;12(2):256. doi: 10.3390/jpm12020256 (PMC8879344; doi:10.3390/jpm12020256)
Supplement: Supplementary file 1 [file jpm-12-00256-s001.zip › Supplementary File 0217.pdf]

Supplementary File

Features of the institutional online SDM process

Figure S1 depicts the features of the institutional online SDM process. In the panel "a" the display of all SDM topics can be found in the hospital information system (HIS) to trigger the SDM process. Checking the SDM button will direct to a dedicated page with a pull-down menu consisting of all of the built-in web-based SDM topics. Confirmed selection of a specified topic will direct to the process page. The clinicians can check for their progress and records and print out an SDM sheet containing a QR code for the patent and surrogates for off-site review and assessment. Panel "b" shows an example of a record for the SDM process. The HIS will initiate an online record process for the face-to-face SDM process and then generate a record among the patient's medical records. The coach or the healthcare team member can also track the responses from the patient and surrogates after they review the patient decision aids (PDA) contents. Panel "c" shows an example of an on-screen display of the QR code, which directs to the online information for the SDM participants. This panel takes the topic "Long-term care settings for ventilator dependence" as an example, with case-sensitive codes shown on screen or print-out PDA contents handled to the patient and surrogates. The PDA was co-designed and customized for each topic but generally contained five steps according to the literature. Panel "d" shows the online self-assessment function, designed with the REDCap structure, provided to the patient and surrogates to help the patient and surrogates find out their preference and value for the options, including the fourth step consisting of six questions for self-assessment regarding the choice, and the fifth step for assessing the preparedness for decision-making. Panel "e" exemplifies the feedback evaluation by the patient and surrogates for the SDM process they participated in. The REDCap system automatically directs to the evaluation page after the patient and surrogate review PDA contents. The SDM team members can be authorized to access the stored data in the REDCap system entered by the responders.

Clinicians' feedback evaluation of the online SDM integration

Table S1 summarizes the results of clinicians' feedback evaluation of the online SDM process based on a customized questionnaire from our institution with random sampling of ten physicians who used the related SDM topics with online process experience. This questionnaire consists of four statements scored on a five-point Likert scale, ranging from 1 (completely disagree) to 5 (completely agree). As shown in this table, the responses considered this online integration of the SDM process saved time, especially for the SDM process. On the other hand, they showed the lowest score regarding the effectiveness of online integration in improving patient care (Table S1).

Table S1. Physicians' response to online integration of the SDM process (n=10).

Item	Data
Online SDM integration reduces the time burden of the medical team members.	4.7±1.1
Online SDM integration significantly reduces the data processing time.	4.4±0.5
Online SDM integration increases the convenience of the medical team.	4.3±0.5
Online SDM integration improves the effectiveness of patient care.	4.1±0.9

The interview examples from the clinical staff are provided below to illustrate the opinions for the system of the SDM integration (translated from the original Chinese text):

"The informational forms fill out online, which is very helpful for saving time and seeing whether the patient has made a decision or not."

"In general, we talk about the healthcare options with patients or patients' family members carefully and individually, but sometimes it will take more time to explain with them, which will be

troublesome for the time for other clinical situations. The information of SDM process helps the patients and their family members take the PDA back home easily and have more time to think or discuss, then fill out the form online to express the decision preparedness."

"With the system, it saves time for me to collect the evaluation data reported from our patients. Let us know the performance of SDM implementation."

"Before the online SDM system was released, the feedback after option interviews from patients were hard to know timely. I was asked to co-design the online REDCap program from the quality management center. I have put some important questions for our care team in our PDA evaluation. And now I can see the responses in the EMR system if they have finished the PDA."

"The patients cannot speak for themselves is one of the characteristics in Pediatrics. Thus, it's important to seek the parents' participation. The online SDM system is a good way to let the parents have more time for discussing and reaching a decision for the treatment options."

Clinicians' evaluation of the whole SDM process

Table S2 summarizes the results of the clinicians' evaluation of the whole SDM process using the Shared Decision Making Questionnaire–physician version (SDM-Q-Doc) consisting of nine statements that scored on a six-point Likert scale [1]. The results suggested that the clinicians placed high scores on the nine questions, with the lowest average scores of "selecting option together" (5.0±1.1) and "agree on how to proceed" (5.1±1.0) (Table S2).

Table S2. Clinicians' evaluation of the whole SDM process (n=197).

Item	Data
I made clear to my patient that a decision needed to be made.	5.3±0.8
I wanted to know exactly from my patient how he/she wants to be involved in making the decision.	5.3±0.8
I told my patient that there are different options for treating his/her medical condition.	5.3±0.7
I precisely explained the advantages and disadvantages of the treatment options to my patient.	5.4±0.8
I helped my patient understand all the information.	5.3±0.8
I asked my patient which treatment option he/she prefers	5.3±0.9
My patient and I thoroughly weighed the different treatment options.	5.3±0.8
My patient and I selected a treatment option together.	5.0±1.1
My patient and I reached an agreement on how to proceed.	5.1±1.0

References

1. Scholl, I.; Kriston, L.; Dirmaier, J.; Buchholz, A.; Härter, M., Development and psychometric properties of the Shared Decision Making Questionnaire–physician version (SDM-Q-Doc). *Patient Edu Couns* **2012,** 88, 284-290.

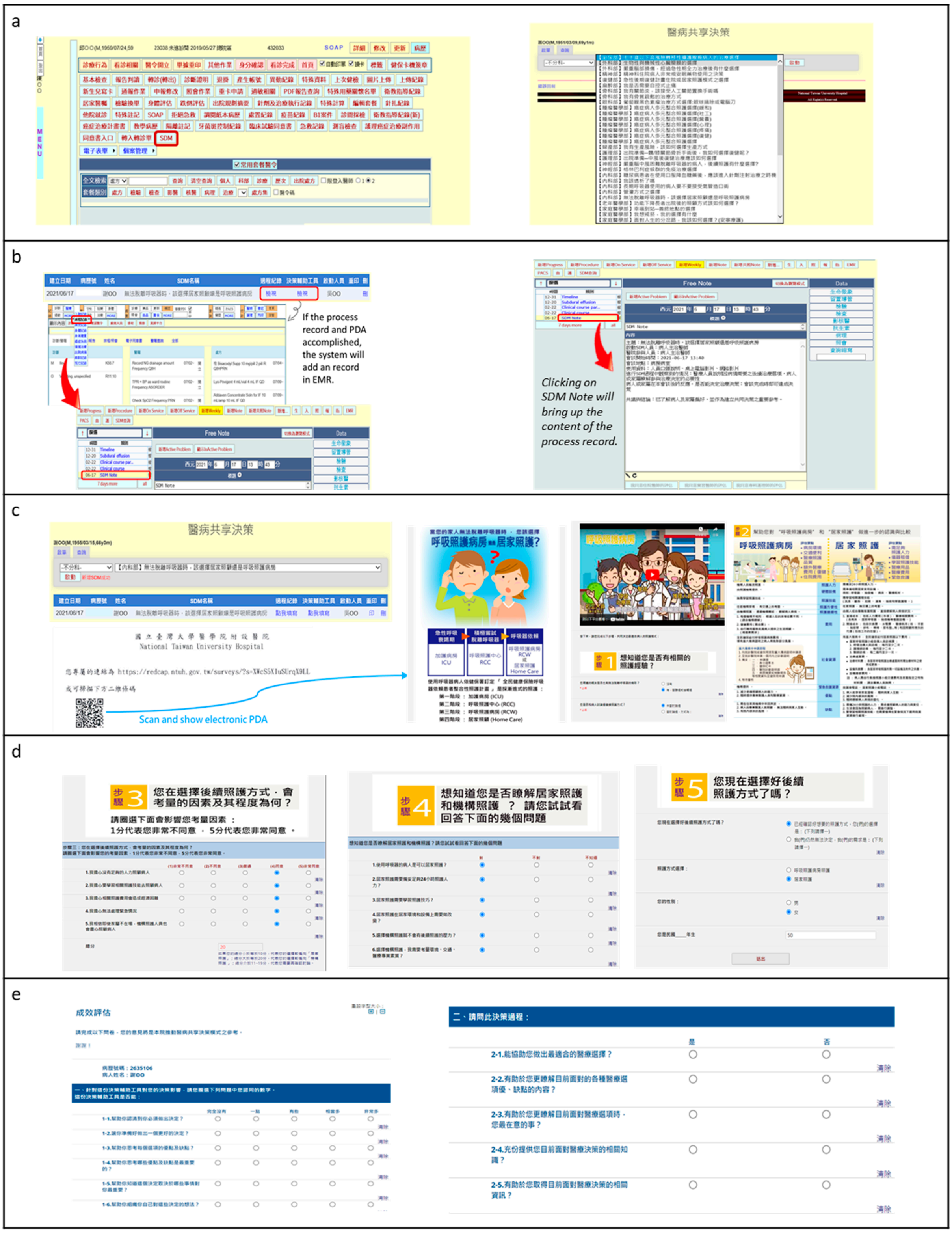

Figure S1. Features of the online SDM process. (a) Display of all topics in the HIS for triggering SDM. (b) Structured records of the SDM process. (c) Online information for the participants of SDM. (d) Self-assessment of the patient/surrogates. (e) Feedback evaluation for the SDM process.
